# Supplementary figures and images for: Cuproptosis-Related lncRNA Gene Signature Establishes a Prognostic Model of Gastric Adenocarcinoma and Evaluate the Effect of Antineoplastic Drugs
Source: Genes (Basel). 2022 Nov 25;13(12):2214. doi: 10.3390/genes13122214 (PMC9777654; doi:10.3390/genes13122214)

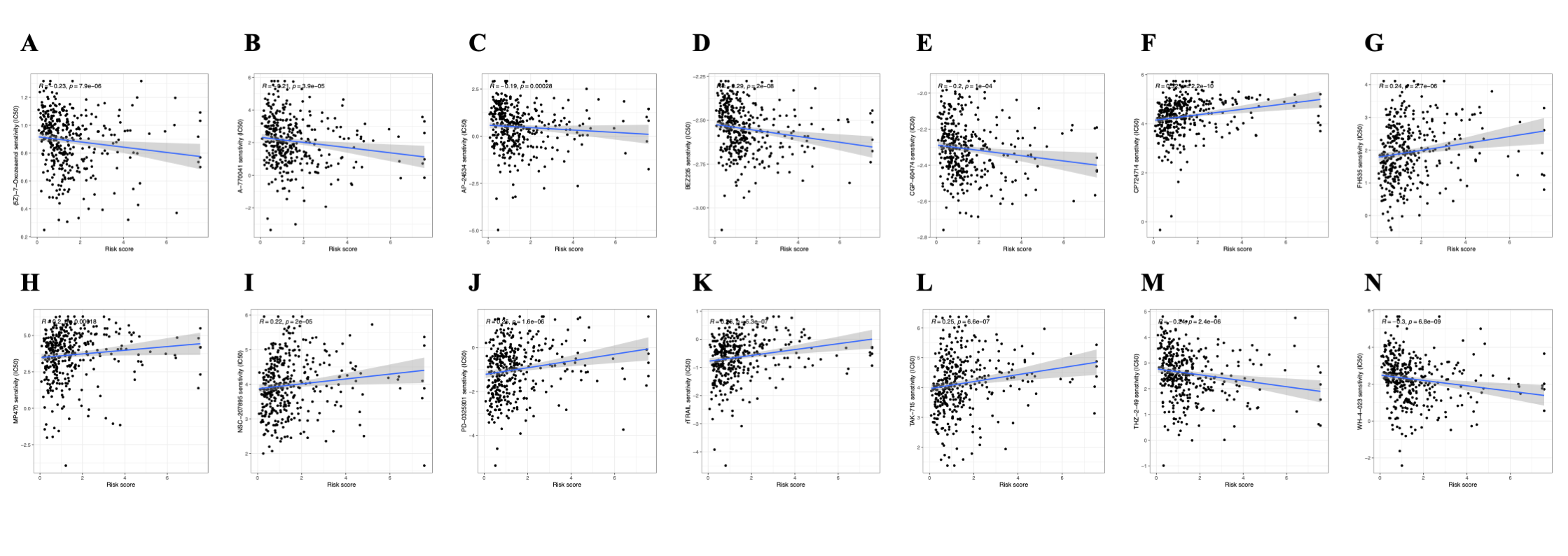

Supplement: Supplementary file 1 [file genes-13-02214-s001.zip › Supplement Figure S1.tiff]

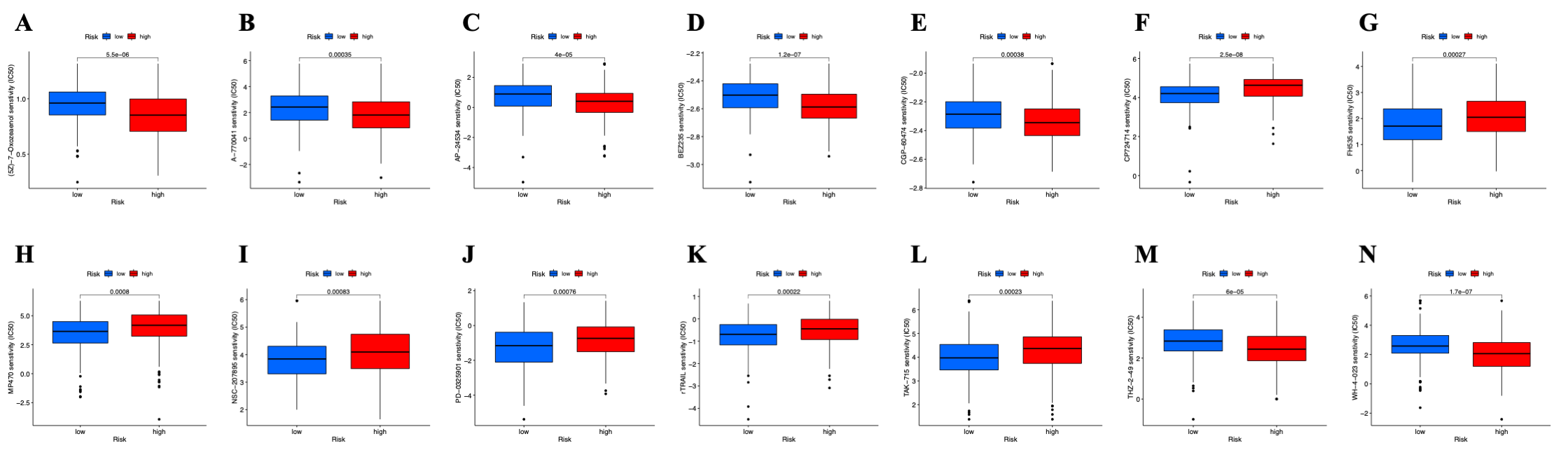

Supplement: Supplementary file 1 [file genes-13-02214-s001.zip › Supplement Figure S2.tiff]
